# Brain morphology changes after spinal cord injury: A voxel-based meta-analysis

**DOI:** 10.3389/fneur.2022.999375

**Published:** 2022-09-01

**Authors:** Haiyang Yu, Duanyong Chen, Hai Jiang, Guangtao Fu, Yuhui Yang, Zhantao Deng, Yuanfeng Chen, Qiujian Zheng

**Affiliations:** ^1^Department of Orthopedics, Guangdong Provincial People's Hospital, Guangdong Academy of Medical Sciences, Guangzhou, China; ^2^Guangdong Cardiovascular Institute, Guangdong Provincial People's Hospital, Guangdong Academy of Medical Sciences, Guangzhou, China; ^3^Research Department of Medical Science, Guangdong Provincial People's Hospital, Guangdong Academy of Medical Sciences, Guangzhou, China; ^4^Department of Orthopedics, Southern Medical University, Guangzhou, China

**Keywords:** spinal cord injury, voxel-based morphometry, brain mapping, neuroimaging, meta-analysis

## Abstract

**Objectives:**

Spinal cord injury (SCI) remodels the brain structure and alters brain function. To identify specific changes in brain gray matter volume (GMV) and white matter volume (WMV) following SCI, we conducted a voxel-based meta-analysis of whole-brain voxel-based morphometry (VBM) studies.

**Methods:**

We performed a comprehensive literature search on VBM studies that compared SCI patients and healthy controls in PubMed, Web of Science and the China National Knowledge Infrastructure from 1980 to April 2022. Then, we conducted a voxel-based meta-analysis using seed-based d mapping with permutation of subject images (SDM-PSI). Meta-regression analysis was performed to identify the effects of clinical characteristics.

**Results:**

Our study collected 20 studies with 22 GMV datasets and 15 WMV datasets, including 410 patients and 406 healthy controls. Compared with healthy controls, SCI patients showed significant GMV loss in the left insula and bilateral thalamus and significant WMV loss in the bilateral corticospinal tract (CST). Additionally, a higher motor score and pinprick score were positively related to greater GMV in the right postcentral gyrus, whereas a positive relationship was observed between the light touch score and the bilateral postcentral gyrus.

**Conclusion:**

Atrophy in the thalamus and bilateral CST suggest that SCI may trigger neurodegeneration changes in the sensory and motor pathways. Furthermore, atrophy of the left insula may indicate depression and neuropathic pain in SCI patients. These indicators of structural abnormalities could serve as neuroimaging biomarkers for evaluating the prognosis and treatment effect, as well as for monitoring disease progression. The application of neuroimaging biomarkers in the brain for SCI may also lead to personalized treatment strategies.

**Systematic review registration:**

https://www.crd.york.ac.uk/prospero/display_record.php?ID=CRD42021279716, identifier: CRD42021279716.

## Introduction

Spinal cord injury (SCI) is a permanent neurological dysfunction including complete or partial loss of motor, sensory and visceral function below the site of injury (1, 2). After spinal cord injury, a series of secondary degenerative changes can spread across the entire neuroaxis from the spinal cord to the brain and continue to progress over the following years (3, 4). Researchers observed apoptotic cell death, decreased dendritic spine density and reduced angiogenesis after SCI that led to significant atrophy in the sensorimotor systems (5–7). These structural changes may be triggered by retrograde degeneration of central motor nerve fibers along the myelinated axons, resulting to the soma shrinkage of corticospinal projecting neurons (8). However, the specific pathophysiological mechanisms have not yet been fully elucidated (9). Recent studies have suggested that brain alterations following SCI are associated with lesion severity and long-term outcomes (8, 10). Thus, the investigation of brain alterations after SCI is of high clinical importance for evaluating injury severity, predicting outcomes and providing personalized treatment strategies (4, 11). However, there is currently a lack of large samples and large-scale in-depth analysis combining brain structure changes and functional prognosis.

Voxel-based morphology (VBM) is an automatic technique that allows accurate comparison of volume differences between groups at the whole-brain level (12). Currently, VBM has become a well-established method for detecting volumetric brain abnormalities in subjects with various psychological disorders, including major depressive disorder, bipolar disorder and schizophrenia (13–15). In recent years, a large number of VBM studies have been performed to capture macrostructural changes between SCI patients and healthy controls (16–18). Some VBM studies reported diverse brain alterations in a wide range of regions, including the corticospinal tract (CST), primary motor cortex, anterior cingulate gyrus and thalamus; however, the results are inconsistent across studies (5, 19, 20). Other VBM studies did not detect any brain alterations in SCI patients relative to the brains of healthy controls (21, 22). Previous VBM study also uncovered that various clinical characteristics including injury level, severity, and duration can affect the brain alterations after SCI (23). In fact, most neuroimaging studies on SCI showed list of limitations including small cohort sizes (<20 patients), differences in patient characteristics (i.e., level and severity of injury), image acquisition, and post processing techniques, which may lead to inconsistent results and statistical inefficiency (4, 24). To better understand the brain alterations and their potential core pathophysiology process following SCI, the mentioned limitations need to be fully considered and quantitative meta-analysis are able to address a numbers of methodological concerns that contribute to varied findings at the study level and allow the identification of reliable and authentic findings in the literature.

Coordinate-based meta-analyses (CBMA) are significant and powerful tools to assess brain abnormalities in voxel-based neuroimaging studies (25, 26). The techniques for CBMA have been evolving and applied in recent years (27, 28). Seed-based d mapping with permutation of subject images (SDM-PSI), the latest generation of the CBMA method, allows a familywise error (FWE) correction for multiple comparisons of the results, which improves statistical efficiency (29). Here, the objective of our study were (1) to identify consistent patterns of brain alterations after SCI as detected by structural brain imaging; (2) to identify the possible correlation between brain volume and clinical characteristics at study level. Therefore, we conducted a meta-analysis of a relatively large sample (more than 20 studies) VBM studies in SCI patients with the latest version of anisotropic effect size seed-based d mapping. We also conducted a meta-regression analysis to evaluate the potentially confounding effect of clinical characteristics in SCI patients.

## Methods

Our study was conducted following the Preferred Reporting Items for Systematic Reviews and Meta-analyses (PRISMA) statement (30) (Supplementary Table 1) and the latest tutorial for CBMA (31).

### Search strategy and study selection

We conduct a comprehensive search of VBM imaging studies (from 1950 to April 2021) focus on gray matter and white matter in electronic databases, including PubMed, Web of Knowledge and China National Knowledge Infrastructure. We use search phase “(Spinal cord injury OR spinal cord injuries) AND (‘morphometry' OR ‘voxel-based' OR ‘voxelwise' OR ‘VBM' OR ‘structural MRI')” to conduct executive research. In addition, we also conducted manual search over the referenced works in reviews. After screening the titles and abstracts, we then carefully viewed full text to determine whether articles meet inclusion/exclusion criteria. We only included all peer-reviewed studies which compared the gray matter volume (GMV) or white matter volume (WMV) between SCI and healthy controls.

The inclusion criteria for quantitative meta-analysis were: (1) reporting comparison between SCI patients and health controls, (2) employing T1 MRI covering the whole brain, (3) performing whole-brain voxel-based comparison of GMV or WMV between SCI patients and health control, (4) reporting coordinates (x, y, z) and height of the peaks (*t*-value, *P* –value, or *z*-value) in Talairach or Montreal Neurological Institute (MNI) space. The exclusion criteria were: (1) non-empirical studies, including reviews, case-reports, conference abstracts, letters, (2) sample size <10, (3) duplicated studies, (4) inclusion of minors. Two authors independently check the eligibility of all identified studies. In case of disagreement, the two researchers reached a consensus through discussion.

### Database construction

After reading the manuscript, we extracted data from the literature that met the inclusion and exclusion criteria. We recorded sample characteristics, including the sample size, age, and sex of SCI patients and healthy controls separately. For SCI patients, we also recorded clinical and behavior data, encompassing the time since injury (TSI), American Spinal Injury Association (ASIA) grade and score (81). For each study, we also recorded statistical information, including the software packages it used, the stereotactic spaces (Talairach or MNI space), the significant statistical level, peak coordinates and height of peaks (*t*-value, *P*-value, or *z*-value). To make the data uniform, we converted the *P*-value or *z*-value into a *t*-value with a statistics converter (https://www.sdmproject.com/utilities/?show=Statistics). To minimize errors, we intended to copy and paste the numbers of interest from the literature rather than manually typing the data into our spreadsheet files. We organized all mentioned data in the table systematically so that SDM-PSI software could read the data. Two authors checked the data independently.

### Quality assessment

To assess the quality of the enrolled articles, a 12-point checklist, including a comprehensive evaluation of demographic characteristics, the sample size, the quality of the reported results, and imaging-specific methodology, was applied (Supplementary Table 2) (31, 32). The checklist provided some objective indicators for each study, and the final quality scores are shown in Table 1.

**Table 1:** Demographic and clinical characteristics of included VBM studies.

**Study**	**Sample size (male)**		**Age, mean (SD)**		**Clinical characteristics**				**Quality scores (12)**
	**Patients**	**Controls**	**Patients**	**Controls**	**TSI (years)**	**MS (SD)**	**PPS (SD)**	**LTS (SD)**	
Jurkiewicz et al. (71)	17 (13)	17 (9)	33.1 (8.9)	28.1 (NA)	4.3	28.3 (16.2)	16.2 (6.7)	15.2 (7.2)	11.5
Lundell et al. (72)	19 (18)	7 (7)	46.1 (12.3)	41.7 (18.1)	13.0	NA	NA	NA	10
Freund et al. (73)	10 (10)	16 (NA)	47.1 (10.7)	39.3 (15.4)	6.9	46.1 (21.2)	66.5 (NA)	96.3 (NA)	12
Freund et al. (8)	13 (10)	18 (12)	46.9 (20.2)	35.0 (9.3)	1.0	59.9 (27.0)	NA	NA	11
Yoon et al. (60)	10 (7)	10 (6)	39.8 (6.2)	39.5 (8.6)	1.5	NA	NA	NA	11
Hou et al. (59)	20 (11)	30 (17)	36.3 (5.6)	35.2 (8.9)	0.2	49.1 (26.5)	46.3 (30.5)	44.1 (29.3)	12
Mole et al. (22)	18 (NA)	18 (NA)	51.3 (NA)	NA	11.1	60.3 (NA)	57.7 (NA)	56.2 (NA)	11.5
Mole et al. (22)	12 (NA)	18 (NA)	54.3 (NA)	NA	17.7	47.8 (NA)	57.5 (NA)	61.9 (NA)	11.5
Villiger et al. (47)	9 (5)	14 (7)	55.1 (15.8)	47.1 (14.4)	1.0	NA	NA	NA	11
Chen et al. (74)	20 (12)	30 (19)	37.7 (13.4)	36.9 (5.2)	NA	NA	NA	NA	10.5
Jiao et al. (75)	13 (7)	15 (8)	26.3 (7.9)	25.7 (5.8)	0.2	36.5 (25.9)	43.3 (27.3)	42.5 (30.3)	12
Jutzeler et al. (76)	30 (27)	31 (23)	46.3 (11.9)	42.1 (9.9)	10.5	63.4 (24.5)	NA	NA	11
Chen et al. (16)	21 (15)	21 (15)	50.5 (12.1)	50.0 (12.4)	5.2	NA	NA	NA	11
Zhou et al. (77)	16 (14)	20 (17)	47.1 (11.7)	47.1 (10.1)	NA	NA	NA	NA	10.5
Seif et al. (78)	24 (19)	23 (13)	49.7 (19.8)	35.9 (10.9)	0.12	NA	NA	NA	11
Ziegler et al. (3)	15 (14)	18 (NA)	47.5 (19.4)	NA	1.1	63.4 (28.0)	55.5 (25.7)	71.2 (29.7)	11.5
Li et al. (17)	19 (12)	19 (NA)	53.4 (12.4)	NA	1.0	78.5 (18.4)	NA	NA	11
Guo et al. (23)	59 (49)	37 (24)	38.2 (11.3)	39.8 (9.0)	2.1	50.3 (17.1)	66.0 (23.6)	64.2 (24.1)	12
Wang et al. (79)	24 (22)	26 (24)	38.5 (11.2)	38.4 (10.6)	0.5	50.9 (4.7)	NA	NA	11.5
Huynh et al. (68)	19 (16)	24 (20)	57.1 (10.2)	51.9 (12.9)	16.3	NA	NA	NA	10.5
Huynh et al. (68)	10 (8)	24 (20)	57.0 (10.7)	51.9 (12.9)	17.4	NA	NA	NA	10.5
Muryama et al. (80)	12 (9)	12 (9)	43.4 (15.0)	38 (12.0)	0.6	NA	NA	NA	10.5

TSI, time since injury; MS, motor score; SS, sensory score; LTSS, light touch score; VBM, voxel-based morphology.

### Meta-analysis method

We used SDM-PSI version 6.21 (https://www.sdmproject.com/) to conduct quantitative meta-analysis (29). GMV and WMV datasets were introduced into SDM-PSI software and analyzed separately following the standard procedure. First, we calculated the map of lower and upper bounds of effect size of each study according to the peak information (correlation template “gray matter” or “white matter” mask, anisotropy = 1, isotropic full width half maximum = 20 mm and voxel size = 2 mm). Second, we estimated the map of the most likely effect size maps and standard errors for each study based on the MetaNSUE algorithms (31). Third, images of imputed effect size maps were combined using a standard random-effects meta-analysis, and the meta-analytic maps were combined following Rubin's rules. Fourth, subject images of each study were imputed, and FWE correction was conducted for multiple comparisons. Finally, statistical significance was assessed using threshold-free cluster enhancement (TFCE) in the statistical thresholding (cluster level, *P* < 0.05, FWE corrected for multiple comparisons and voxel extent ≥ 10).

### Reliability analysis and subgroup meta-analysis

To assess the replicability of the results, we conducted jackknife sensitivity analysis for both GMV and WMV datasets by repeating the meta-analysis after leaving out one study each time (33). Then, we conducted subgroup meta-analysis to evaluate potential confounding effects. Variables including TSI > 1 year was assessed. The TFCE-based FWE corrected threshold (cluster level, *P* < 0.05, FWE corrected for multiple comparisons and voxel extent ≥ 10) was used to determine statistical significance.

### Meta-regression model

We conducted a meta-regression model to investigate the possible associations between brain volumes (GMV or WMV) and clinical characteristics, including TSI, injury severity (percentage of complete SCI) and ASIA motor score. However, clinical data extracted from the literature are at the study level and are unable to provide information about specific patients. Thus, we conducted permutation at the study level for meta-regression and used the Freedman-Lane procedure for optimal statistical properties. Statistical significance was determined using a threshold of *P* < 0.05 and cluster extent ≥ 10 voxels in meta-regression analyses.

### Heterogeneity and publication bias

To test heterogeneity and publication bias, we selected a peak which show the largest cluster of voxels from the Meta-analysis. Then we extracted the mean value to test heterogeneity *I*^2^ statistic where *I*^2^ <50% suggests low heterogeneity and created a funnel plot and Egger tests to check publication bias (33). An asymmetric plot and *P* < 0.05 were considered statistically significant.

## Results

### Selected studies and sample character

**Figure 1:**
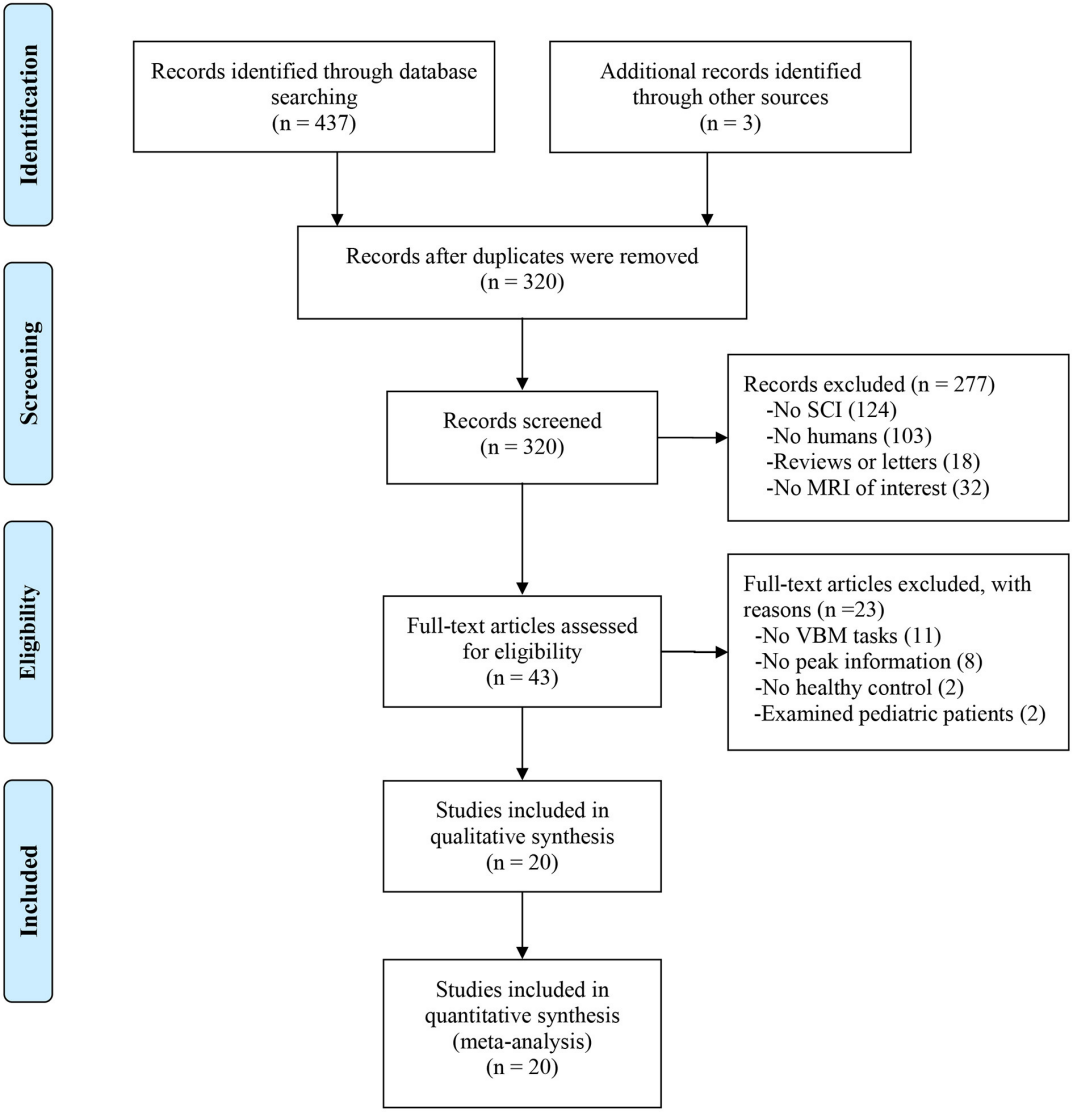
Flow diagram of inclusion and exclusion process of selected VBM studies. SCI, spinal cord injury; VBM, voxel-based morphology.

After screening the abstract and viewing the full text, we included 20 structural MRI studies in our meta-analysis that comprised 410 SCI patients and 406 healthy controls (see Figure 1). All studies reported balanced age and sex distributions between patients and healthy controls, while four datasets did not report the mean age and sex of healthy controls.

The clinical characteristics of the participants and the quality of each study are shown in Table 1. The quality of the included studies was acceptable.

### Meta-analysis of GMV and WMV

**Figure 2:**
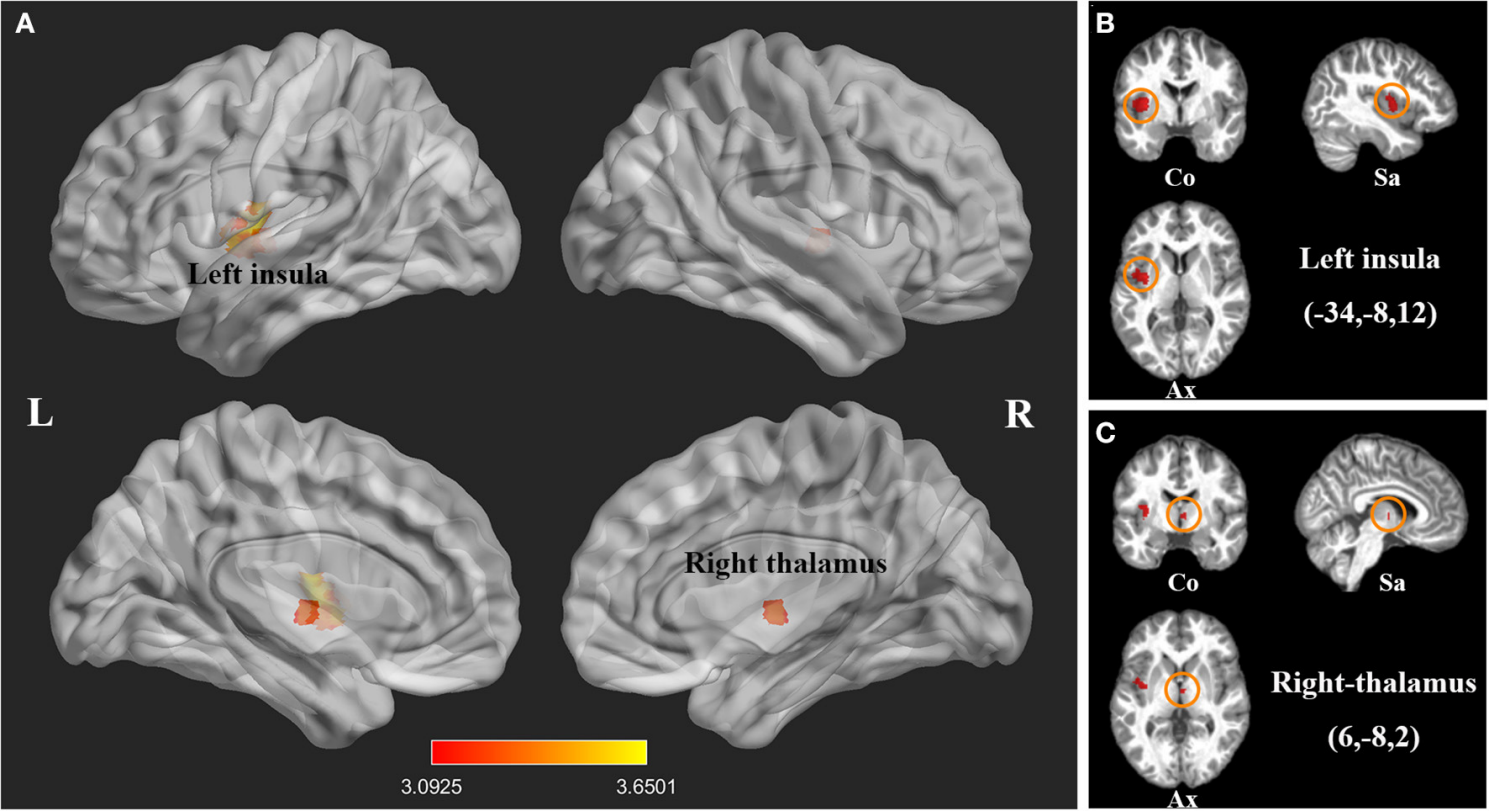
Statistical parametric maps showing decreased gray matter volumes in patients with spinal cord injury (SCI) compared with those of healthy controls in the pooled meta-analysis. **(A)** The overlay of statistical parametric maps showing the decreased gray matter volume in left insula and right thalamus in SCI patients compared with healthy controls. **(B)** The illustration of the sagittal, coronal and axial planes of the decreased gray matter volume in left insula in SCI patients compared with healthy controls. **(C)** The illustration of the sagittal, coronal and axial planes of the decreased gray matter volume in right thalamus in SCI patients compared with healthy controls. Color bar represents *t*-value; Co, Sa, and Ax represent coronal, sagittal and axial planes, respectively. R, right; L left, spinal cord injury, SCI.

We investigated 20 GMV datasets (410 SCI patients and 406 health controls). Compared with controls, SCI patients displayed significant gray matter atrophy in two clusters, which peaked in left insula (FWE-corrected for multiple comparisons *P* < 0.001, *z* = −3.65) and right thalamus (FWE-corrected for multiple comparisons *P* < 0.001, *z* = −3.51). The cluster of right thalamus also extended to left thalamus (Table 2, Figure 2).

**Table 2:** Regional differences in gray matter and white matter volume between SCI patients and healthy controls.

**Region**	**MNI, x, y, z**	**SDM-Z**	***P*** **-value**	**Voxels**	**Cluster breakdown** **(No. of voxels)**	**Jackknife sensitivity**
**GM (Patients < Controls)**						
Left insula, BA 48	−34, −8, 12	−3.65	<0.001	134	Left insula, BA 48	22/22
					Left rolandic operculum, BA 48	
Right thalamus	6, −8, 2	−3.51	<0.001	34	Right thalamus	22/22
**WM (Patients < Controls)**						
Right cortico-spinal projections	4, −42, −60	−5.00	<0.001	292	Right cortico-spinal projections	14/4
					Left cortico-spinal projections	

GM, gray matter; WM, white matter; MNI, Montreal Neurological Institute; SDM, signed differential mapping; FWE-corrected P < 0.05, and voxel extent ≥ 10.

**Figure 3:**
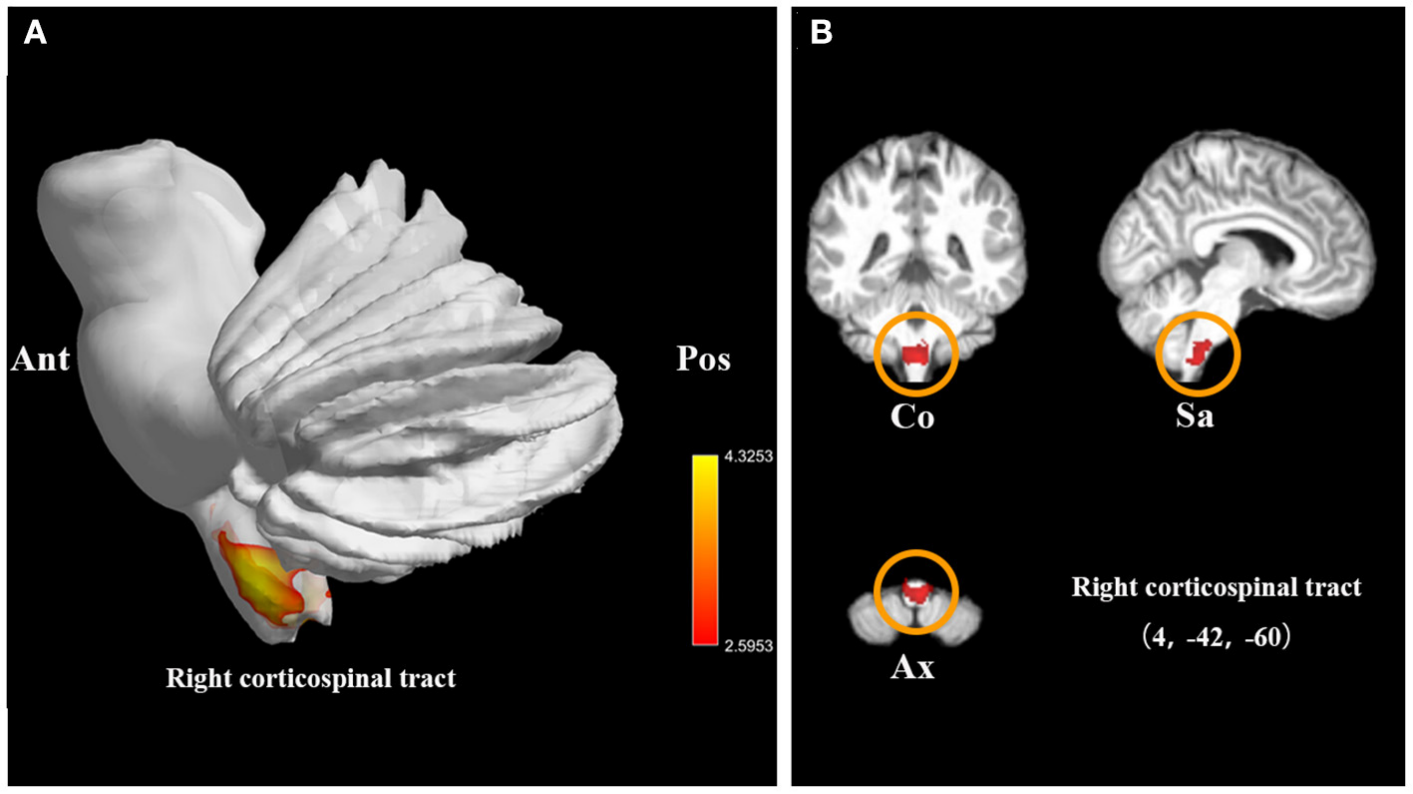
Statistical parametric maps showing the decreased white matter volumes in patients with spinal cord injury (SCI) compared with those of healthy controls in the pooled meta-analysis. **(A)** The overlay of statistical parametric maps showing the decreased white matter volume in right corticospinal tract in SCI patients compared with healthy controls. **(B)** The illustration of the sagittal, coronal, and axial planes of the decreased white matter volume in right corticospinal tract in SCI patients compared with healthy controls. Color bar represents *t*-value; Co, Sa, and Ax represent coronal, sagittal, and axial planes, respectively. Ant, anterior; Pos, posterior; SCI, spinal cord injury.

We also investigated 14 WMV datasets (246 SCI patients and 283 health controls). Compared with controls, SCI patients displayed significant WMV loss, which peaked in right CST (FWE-corrected for multiple comparisons *P* < 0.001, *z* = −5.00). The cluster extended to left CST (Table 2, Figure 3).

### Reliability and subgroup analyses

The jackknife sensitivity analysis revealed that gray matter atrophy was highly replicable in all 22 combinations of studies (Table 2), and white matter atrophy in the CST was replicable in all 14 included studies (Table 2).

**Figure 4:**
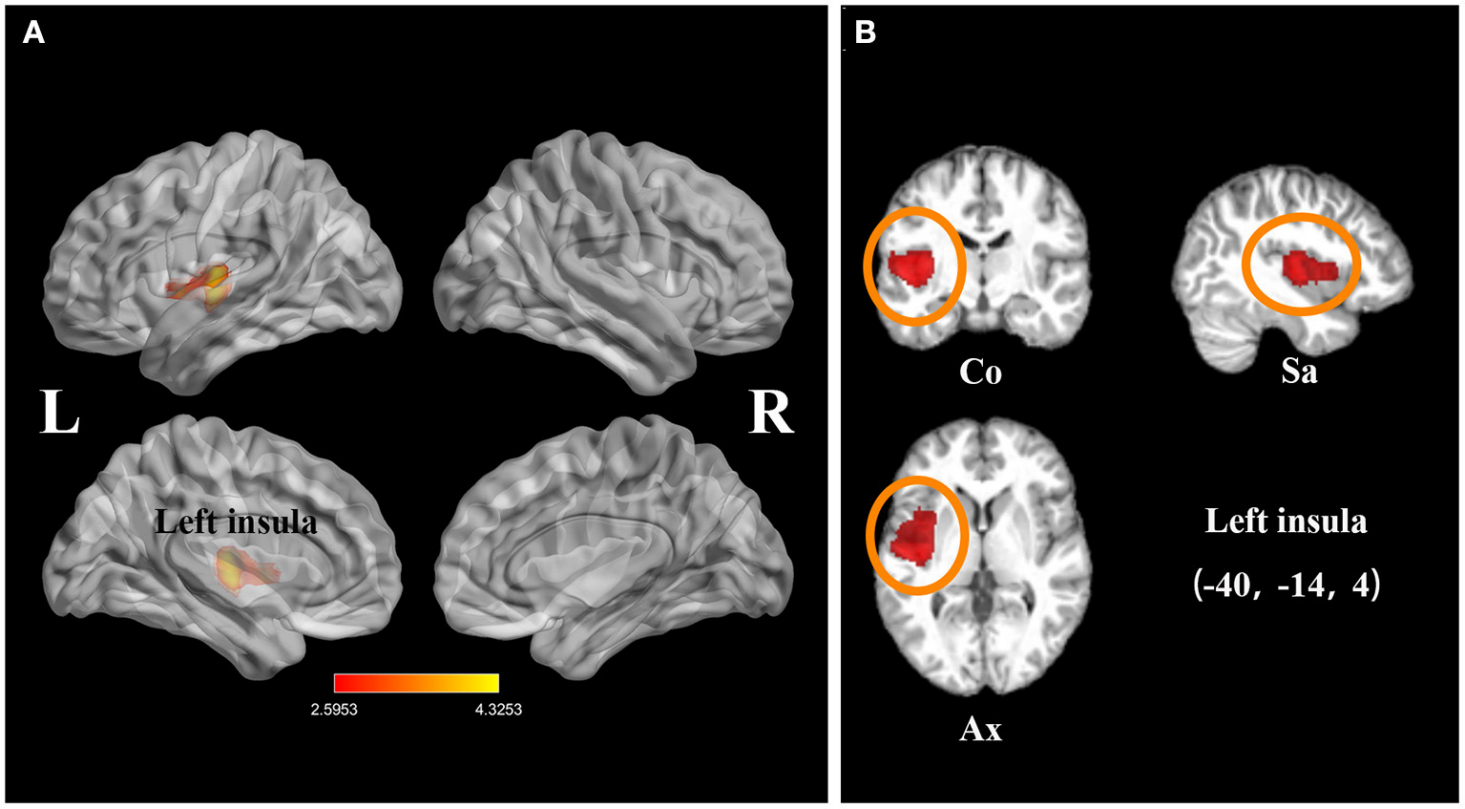
Statistical parametric maps showing the decreased gray matter volumes in patients with spinal cord injury (SCI; time since injury > 1 year) compared with those of healthy controls in the subgroup meta-analysis. **(A)** The overlay of statistical parametric maps showing the decreased gray matter volume in left insula in SCI patients (time since injury > 1 year) compared with healthy controls. **(B)** The illustration of the sagittal, coronal, and axial planes of the decreased gray matter volume in left insula in SCI patients (time since injury > 1 year) compared with healthy controls. Color bar represents *t*-value; Co, Sa, and Ax represent coronal, sagittal, and axial planes, respectively. R, right; L left; SCI, spinal cord injury.

The subgroup analysis of long-term SCI participants (TSI > 1 year) included 14 datasets comprising 262 chronic SCI patients and 231 healthy controls. This analysis revealed that gray matter atrophy in the left insula remained in SCI patients (FWE-corrected for multiple comparisons *P* < 0.001, *z* = −4.33, Table 3, Figure 4).

**Table 3:** Regional differences in gray matter volume between chronic SCI patients and healthy controls.

**Region**	**MNI, x, y, z**	**SDM-Z**	***P*** **-value**	**Voxels**	**Cluster breakdown (No. of voxels)**
Left insula, BA 48	−40, −14, 4	−4.33	<0.001	1,433	Left insula, BA 48

MNI, Montreal Neurological Institute; SDM, signed differential mapping; FWE-corrected P < 0.05, and voxel extent ≥ 10.

### Meta-regression analysis

**Figure 5:**
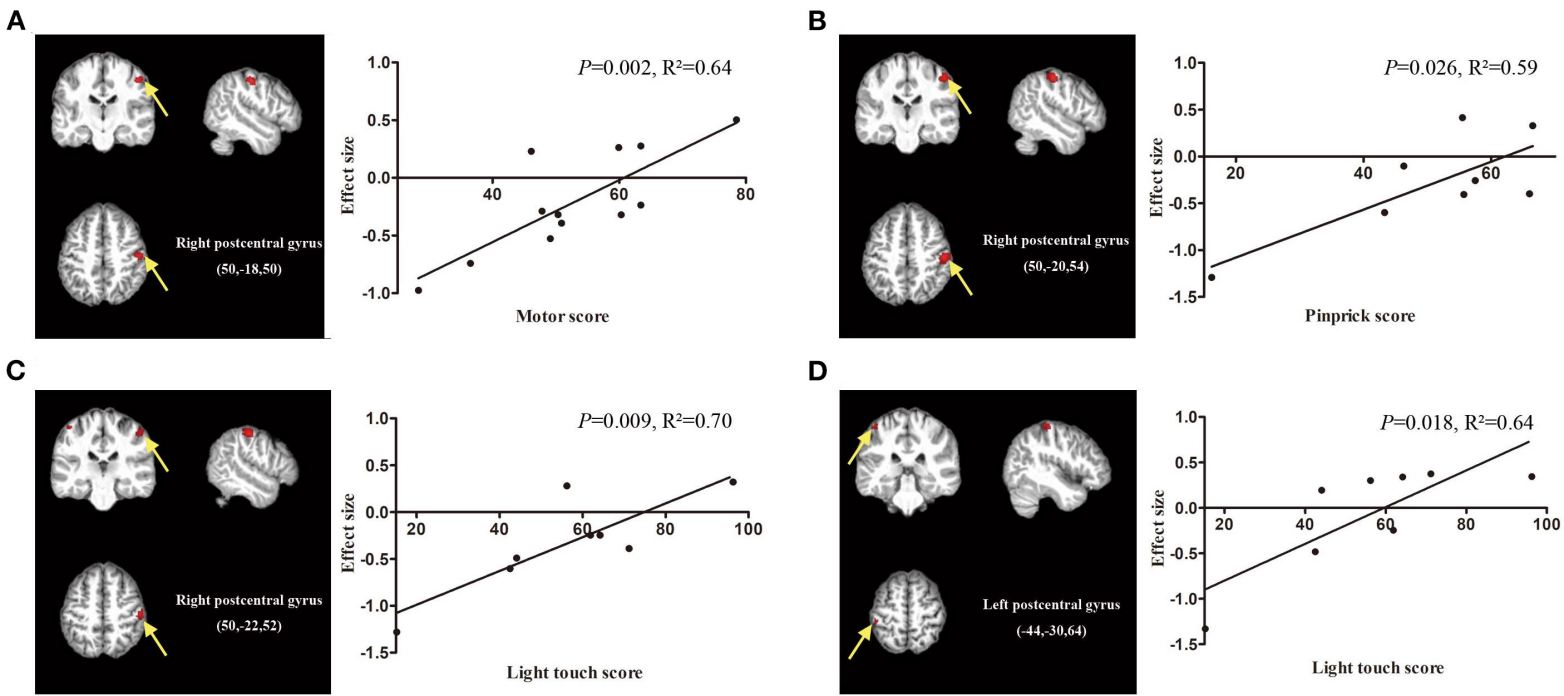
Meta-regression analysis showing the correlation between clinical score and gray matter volume in patients with spinal cord injury (SCI). **(A)** The American Spinal Injury Association (ASIA) motor scores of SCI patients are positively correlated with gray matter volumes in the right postcentral gyrus (*P* = 0.002, *R*^2^ = 0.64). **(B)** The ASIA pinprick scores of SCI patients are positively related to gray matter volumes in the right postcentral gyrus (*P* = 0.026, *R*^2^ = 0.59). **(C)** The ASIA light touch scores of SCI patients are positively related to gray matter volumes in the right postcentral gyrus (*P* = 0.009, *R*^2^ = 0.70) and **(D)** left postcentral gyrus (*P* = 0.018, *R*^2^ = 0.64). The effect sizes in the graph were extracted from the peak of maximum slope significance. Each study is represented as a dot.

Age and TSI data were available in all datasets, ASIA motor scores were available in 14 datasets, and ASIA pinprick scores and light touch scores were available in 8 datasets. Meta-regression analysis showed that the ASIA motor score was positively related to the GMV in the right postcentral gyrus (*P* = 0.002, *R*^2^ = 0.64, Figure 5A). The ASIA pinprick score was positively related to the GMV in the right postcentral gyrus (*P* = 0.026, *R*^2^ = 0.59, Figure 5B). A higher ASIA light touch score of SCI patients was positively related to greater GMV in the right postcentral gyrus (*P* = 0.009, *R*^2^ = 0.70, Figure 5C) and left postcentral gyrus (*P* = 0.018, *R*^2^ = 0.64, Figure 5D). The age of SCI patients was positively related to the GMV in the right precentral gyrus (*P* < 0.001, *R*^2^ = 0.50, Supplementary Figure 1), right supplementary motor area (*P* = 0.002, *R*^2^ = 0.25, Supplementary Figure 1) and left precentral gyrus (*P* = 0.008, *R*^2^ = 0.30, Supplementary Figure 1).

### Heterogeneity

In this study, we calculated *I*^2^ to test the heterogeneity of our results. The *I*^2^-values of the GMV and WMV datasets of SCI patients were 13.3 and 7.3%, respectively, which suggests that the heterogeneity of our study is acceptable (Supplementary Table 3).

## Discussion

To our knowledge, this is the first voxel-based meta-analysis using SDM-PSI to assess brain morphology changes in patients after SCI compared to healthy controls. Our study revealed secondary neural degeneration processed in the motor and sensory pathway and limbic system in SCI patients. The pooled meta-analysis identified reduced GMV in the bilateral thalamus and reduced WMV in bilateral corticospinal projections. In the limbic system, both pooled and subgroup analyses revealed reduced GMV in the insula. Meta-regression analysis revealed that the motor score was positively correlated with the GMV in the right postcentral gyrus, whereas the light touch score was positively related to the GMV in the bilateral postcentral gyrus.

Our study demonstrated gray matter atrophy in the bilateral thalamus, which was in accordance with previous VBM studies and meta-analyses (23, 34–36). Although neurons in the brain are not disrupted directly following SCI, the afferent motor pathway and efferent sensory pathway undergo degenerative processes (37, 38). The thalamus is an important relay station in which the ventral posterior nucleus receives sensory information from the lemniscus medial and spinothalamic tracts and projects to the primary sensory cortex (39–41). A previous study revealed that SCI patients showed a decreased thalamic volume, which was accompanied by iron accumulation and myelin decrease, in comparison with healthy controls 1 year after injury (3). Some researchers have proposed that oxidative stress and chronic inflammation may trigger the breakdown of myelin, leading to iron release (42, 43). However, a recent study discovered that the surface area contracts in the inferior part (representing the lower limb) of the bilateral motor thalamus nucleus, while that of the superior part of the sensorimotor thalamus nucleus (representing the upper limb) expands (44). The decreased local volume of areas representing the lower limb may result from neurodegenerative changes, including transsynaptic degeneration, neural death, demyelination and changes in glial cells (5, 6, 45). The increased local volume of areas representing the upper limb could be attributed to activity-dependent plasticity and axon sprouting after functional training and rehabilitation (46, 47). Therefore, the alteration of the thalamus is a complex process involving both neurodegenerative processes and activity-dependent plasticity.

White matter atrophy was observed in bilateral CSTs at the level of the brainstem, which was consistent with previous meta-analyses and VBM studies (22, 35, 48–50). The CST originates from the sensorimotor cortex and terminates in the motor neurons in the spinal cord, and it is involved in the voluntary movement of limbs and trunks (51, 52). The volume of the CST decreased evidently within the first 40 days after injury (8), which corresponded to Wallerian degeneration in the spinal cord, including axonal degeneration and demyelination in animal models and postmortem human studies (53–55). Specifically, 6 months after injury, the volume of the CST at the brainstem level was associated with long-term recovery following SCI, and the magnitude of CST degeneration, including atrophy and demyelination, in chronic SCI patients was associated with clinical impairment (3, 56). Although degeneration of the CST was confirmed by *in vivo* and animal studies, the CST at the brainstem level may be altered due to activity-dependent plasticity (46, 57, 58). Chronic incomplete SCI patients showed better performance after lower limb training as well as increased brainstem volume (47). In summary, white matter atrophy at the brainstem level can be an alternative target for monitoring treatment effects.

In the present study, no volumetric atrophy was reported in the sensorimotor cortex, while other reviews and VBM studies have reported gray matter atrophy in the primary motor cortex, primary sensory cortex and supplementary motor area following SCI (19, 35). In longitudinal studies, Freund et al. reported gray matter atrophy accompanied by demyelination and iron deposition in the motor cortex and sensory cortex 12 months following injury (8). Hou et al. observed that SCI subjects showed GMV atrophy in the primary motor cortex, primary sensory cortex and supplementary motor area in the early stage of SCI (34, 59). However, the present study did not report volumetric changes in the sensorimotor cortex, which was in accord with several studies (16, 21, 60). The presence of numerous collateral connections in the motor cortex and the connections between the motor cortex and higher-order motor areas may maintain cellular activity, which may reduce or prevent corticospinal neuronal atrophy after SCI (61, 62). Moreover, most SCI patients receive rehabilitation training, and the activity-dependent reorganization after functional training may compensate for the volume decrease in the sensorimotor cortex following SCI (62, 63, 82). To date, the possible mechanisms for cortical alterations have still not been thoroughly investigated in humans. Future imaging studies should provide more information regarding the pathological processes after SCI. Although our study did not report a volume decrease in the sensorimotor cortex, our meta-regression analysis indicated that the volume of the right primary somatosensory cortex was positively related to the motor score and pinprick score, while the bilateral somatosensory cortex was positively associated with the light touch score. Similarly, Guo et al. observed that GMV in the bilateral primary somatosensory cortex was associated with the total motor score and sensory score, which suggested that better clinical performance was associated with greater GMV in the primary somatosensory cortex (23). Therefore, the GMV of the primary somatosensory cortex can assess the extent of neural damage, and a better understanding of primary somatosensory cortex alterations can act as an endpoint to track the treatment effects and identify potential therapeutic agents for developing novel rehabilitation therapies.

Our study revealed significant gray matter atrophy in the left insula after SCI. The GMV reduction in the insula may result from complications following SCI, including mental disorders and neuropathic pain. The insula acts as an important hub that connects the executive system and limbic systems and is involved in limbic integration, psychiatric disorders, motor control, and pain and temperature sensation (64). As an important part of the limbic system, the posterior portion of the insula receives pain input from the spinothalamocitonic circuit, which plays a crucial role in pain processing and modulation (65). Compared with healthy controls, patients with neuropathic pain showed consistently decreased GMV in the bilateral insula (66, 67). In SCI patients with a greater extent of neuropathic pain, stronger functional connectivity of the insula and thalamic subregions has been identified to be associated with a greater intensity of neuropathic pain (67). However, Huynh et al. reported that patients without neuropathic pain showed decreased GMV in the left insula compared to that of patients with neuropathic pain (68). Therefore, the alterations in the insula in SCI patients may not only be driven by pain. Previous morphometric studies have revealed that alterations of the insula are linked to anxiety and depression disorder, and the thickness of the left insula has suggested to be associated with the risk of major depression disorder in middle-aged adults (69). In functional MRI studies, insular activity was significantly decreased in patients with major depressive disorder (70). Thus, the reduced volume of the insula may partly explain the high occurrence of depression disorder after SCI (35). In the subgroup analysis, chronic SCI patients (TSI > 1 year) also showed GMV reduction in the insula, which indicates that the mental or pain-related morphology changes can be long lasting. In brief, GMV atrophy in the left insula might provide potential biomarkers for mental disorder diagnosis after SCI. In the future, more attention should be given to the structural and functional relations of changes in the insula with mental disorders and neuropathic pain following SCI.

Our study had some limitations. First, our study only assessed the macrostructural characteristics of brain atrophy following SCI and did not detect and quantify the microstructural features of trauma-induced pathological changes that reflect widespread demyelination and iron deposition. According to our literature search, the number of quantitative MRI studies using diffusion imaging tensor and relaxation mapping techniques were both <10, which did not meet the CBMA inclusion criteria. Second, the sample size in the subgroup analysis and meta-regressions was not exceptionally large. We performed a comprehensive literature search, and the sample size of the present study was larger than that of a previous meta-analysis. Third, our study only included published VBM studies with peak and coordinates of data, which may lead to selection bias. To assess publication bias, we tested the *I*^2^, which was acceptable. Fourth, voxel-based meta-analysis was conducted according to summarized coordinates and peaks from published literature rather than based on raw image data, which may limit the accuracy of the results. We failed to quantitatively calculate the volume of brain in SCI participants and healthy controls. Finally, our meta-regression analysis only included few clinical characteristics at study level. The studies regarding recovery intervention or neurological disorders was inadequate for conducting meta-regression analysis (sample size <10).

In conclusion, our study demonstrated that SCI comprised the structure of the brain, leading to anatomical atrophy in thalamic-spinal pathways and in the left insula. Neurodegeneration changes in the sensory and motor pathway may result in atrophy in the bilateral thalamus and CST. Furthermore, the high occurrence of mental disorders and neuropathic pain in SCI patients may explain the atrophy in the left insula. In addition, the ASIA clinical score in SCI patients was positively correlated with the volume of the sensorimotor cortex at study level. Our findings suggest that alterations in the brain could contribute to a further understanding of pathological mechanisms and provide essential neuroimaging biomarkers for monitoring mental status and predicting long-term outcomes following SCI. The application of neuroimaging biomarkers in clinical practice could facilitate the development of personalized treatment and nursing.

## Data availability statement

The original contributions presented in the study are included in the article/Supplementary material, further inquiries can be directed to the corresponding authors.

## Author contributions

HY and DC proposed the idea and drafted the manuscript. HY, DC, and YY assisted literature retrieval and filtration and helped manuscript revision. HY, HJ, GF, and ZD drew the illustrations and assisted manuscript polish. YC and QZ were responsible for supervising. All authors contributed to the article and approved the submitted version.

## Funding

The work was partially supported by National Natural Science Foundation of China (32000958).

## Conflict of interest

The authors declare that the research was conducted in the absence of any commercial or financial relationships that could be construed as a potential conflict of interest.

## Publisher's note

All claims expressed in this article are solely those of the authors and do not necessarily represent those of their affiliated organizations, or those of the publisher, the editors and the reviewers. Any product that may be evaluated in this article, or claim that may be made by its manufacturer, is not guaranteed or endorsed by the publisher.

## Abbreviations

ASIA: American Spinal Injury Association
CBMA: coordinate-based meta-analyses
CST: corticospinal tract
FWE: familywise error
GMV: gray matter volume
MNI: Montreal Neurological Institute
PRISMA: preferred reporting items for systematic reviews and meta-analyses
SCI: spinal cord injury
SDM-PSI: Seed-based d mapping with permutation of subject images
TFCE: threshold-free cluster enhancement
TSI: time since injury
WMV: white matter volume
VBM: voxel-based morphology.
