# Supplementary material for: Brain morphology changes after spinal cord injury: A voxel-based meta-analysis
Source: Front Neurol. 2022 Sep 1;13:999375. doi: 10.3389/fneur.2022.999375 (PMC9477418; doi:10.3389/fneur.2022.999375)
Supplement: Supplementary file 2 [file Table_2.DOCX]

# TableS2. Imaging Methodology Quality Assessment Checklist

|  |
| --- |
| **Category 1: Subjects Score (0/0.5/1)** |
| 1 Patients were evaluated prospectively, specific diagnostic criteria were applied, and demographic data was reported |
| 2 Healthy comparison subjects were evaluated prospectively, psychiatric and medical illnesses were excluded |
| 3 Important variables (e.g. age, gender, illness duration, onset time, medication status, comorbidity, severity of illness) were checked, either by stratification or statistically |
| 4 Sample size per group > 10 |
| **Category 2: Methods for image acquisition and analysis** |
| 5 Magnet strength at least 1.5T |
| 6 MRI slice-thickness≤3 mm |
| 7 Whole brain analysis was automated with no a-priori regional selection |
| 8 Coordinates reported in a standard space |
| 9 The imaging technique used was clearly described so that it could be reproduced |
| 10 Measurements were clearly described so that they could be reproduced |
| **Category 3: Results and conclusions** |
| 11 Statistical parameters for significant, and important non-significant, differences were provided |
| 12 Conclusions were consistent with the results obtained and the limitations were discussed |
| **TOTAL /12** |
