# Supplementary material for: Brain morphology changes after spinal cord injury: A voxel-based meta-analysis
Source: Front Neurol. 2022 Sep 1;13:999375. doi: 10.3389/fneur.2022.999375 (PMC9477418; doi:10.3389/fneur.2022.999375)
Supplement: Supplementary file 3 [file Table_3.DOCX]

**TableS3. Heterogeneity of the meta-analysis**

The I^2^ extract from left insula for gray matter volume meta-analysis

| Map | Estimate | Variance | *SDM-Z* |
| --- | --- | --- | --- |
| Hedges' *g* | -0.137495 | 0.010474 | -1.277567 |
| uncorrp | 0.197847 | NA | NA |
| uncorrp_neg | 0.802153 | NA | NA |
| MyTest_*τ*^2^ | 0.026318 | NA | NA |
| MyTest_*H*^2^ | 1.226798 | NA | NA |
| MyTest_*I*^2^ | 13.289492 | NA | NA |
| MyTest_*Q*^2^ | 18.691292 | NA | -0.358323 |

The I^2^ extract from cortico-spinal projections for white matter volume meta-analysis

| Map | Estimate | Variance | SDM-Z |
| --- | --- | --- | --- |
| Hedges' g | -0.142935 | 0.014557 | -1.156123 |
| uncorrp | 0.237304 | NA | NA |
| uncorrp_neg | 0.762696 | NA | NA |
| MyTest_τ2 | 0.014073 | NA | NA |
| MyTest_H2 | 1.110993 | NA | NA |
| MyTest_I2 | 7.317306 | NA | NA |
| MyTest_Q2 | 10.554283 | NA | -0.659161 |
