# Supplementary material for: Brain morphology changes after spinal cord injury: A voxel-based meta-analysis
Source: Front Neurol. 2022 Sep 1;13:999375. doi: 10.3389/fneur.2022.999375 (PMC9477418; doi:10.3389/fneur.2022.999375)
Supplement: Supplementary file 4 [file Data_Sheet_1.DOC]

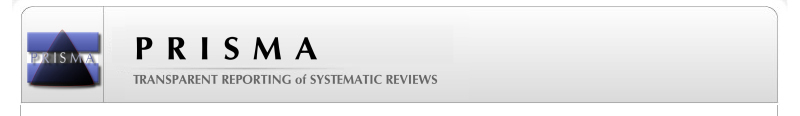
**Error: Reference source not foundPRISMA 2009 Flow Diagram**

**Screening**

**Included**

**Eligibility**

**Identification**

Records identified through database searching
(n = 437)

Additional records identified through other sources
(n = 3)

Records after duplicates were removed
(n = 320)

Records screened
(n = 320)

Records excluded (n = 277)

-No SCI (124)

-No humans (103)

-Reviews or letters (18)

-No MRI of interest (32)

Full-text articles assessed for eligibility
(n = 43)

Full-text articles excluded, with reasons (n =19)

-No VBM tasks (10)

-No peak information (7)

-No healthy control (1)

-Examined pediatric patients (1)

Studies included in qualitative synthesis
(n = 20)

Studies included in quantitative synthesis (meta-analysis)
(n = 20)
