# Supplementary figures and images for: Brain morphology changes after spinal cord injury: A voxel-based meta-analysis
Source: Front Neurol. 2022 Sep 1;13:999375. doi: 10.3389/fneur.2022.999375 (PMC9477418; doi:10.3389/fneur.2022.999375)

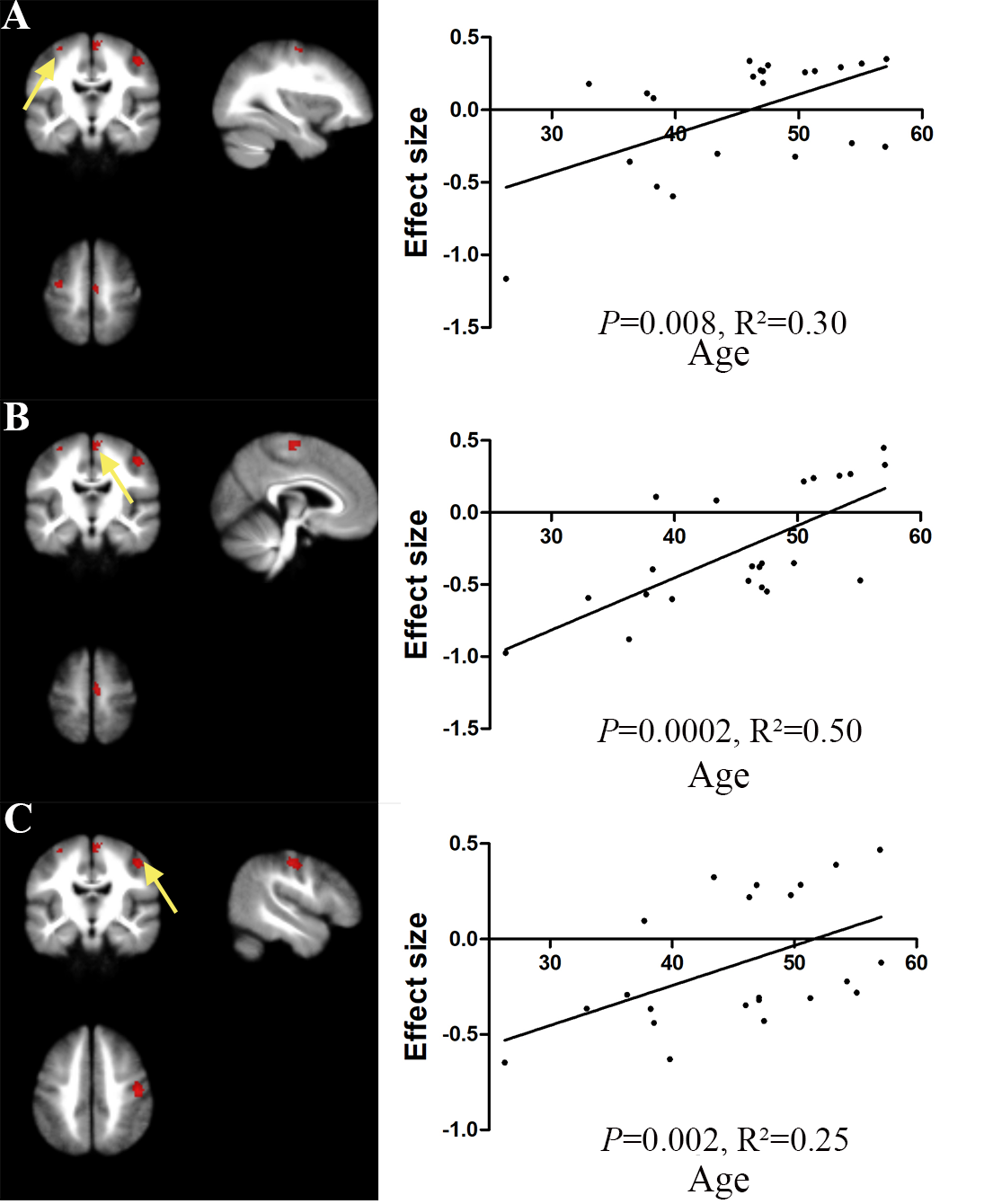

Supplement: Supplementary file 5 [file Image_1.TIF]
